# Ovarian tumorB1-mediated heat shock transcription factor 1 deubiquitination is critical for glycolysis and development of endometriosis

**DOI:** 10.1016/j.isci.2022.105363

**Published:** 2022-10-14

**Authors:** Xi Ling, Jiayi Lu, Xiaoyun Wang, Lan Liu, Lu Liu, Yadi Wang, Yujun Sun, Chune Ren, Chao Lu, Zhenhai Yu

**Affiliations:** 1 Department of Reproductive Medicine, Affiliated Hospital of Weifang Medical University, Weifang 261000, China; 2 School of Clinical Medicine, Weifang Medical University, Weifang 261000, China

**Keywords:** Molecular physiology, molecular biology

## Abstract

Endometriosis is a common chronic condition characterized by abnormal growth of the endometrium outside the uterus. Heat shock transcription factor 1 (HSF1) is a significant regulator of the proteotoxic stress response and plays an essential role in developing endometriosis. However, the mechanisms regulating HSF1 protein stability in endometriosis remain unclear. Here, we demonstrate that OTUB1 interacts with HSF1 and promotes HSF1 protein stability through deubiquitination. In addition, OTUB1 enhances glycolysis and epithelial-mesenchymal transition of endometriosis cells, leading to promote proliferation, migration, and invasion of endometriosis cells. The progression of endometriosis is inhibited in an OTUB1-knockout mouse model. In summary, OTUB1 promotes the development of endometriosis by up-regulating HSF1. OTUB1/HSF1 axis may become a new therapeutic target for endometriosis.

## Highlights

• OTUB1 interacts with HSF1 and promotes HSF1 protein stability via deubiquitination
• OTUB1 enhances glycolysis and EMT of endometriosis cells
• Knockdown of OTUB1 inhibits the development of endometriotic tissue *in vivo*
• OTUB1/HSF1 axis may become a new therapeutic target for endometriosis

## Graphical abstract

**Figure:**
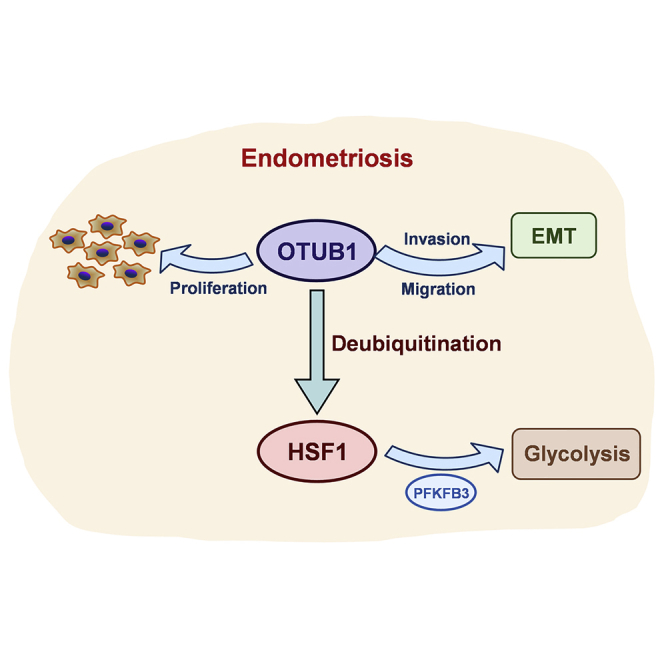

## Introduction

Endometriosis is a chronic gynecologic inflammatory disease characterized by the presence of endometrial tissue outside the uterus, including the ovaries, ligaments, peritoneal surfaces, and intestines (Zondervan et al., 2018). Endometriosis is associated with pelvic pain and infertility, affecting 5-10% of women of childbearing age (Taylor et al., 2021). Currently, the most common treatments for endometriosis are surgery and medications, but these are ineffective for many patients and carry a high risk of recurrence, causing physical and psychological pain (Chen et al., 2022; Murakami et al., 2022). Therefore, there is an urgent need to identify the causes of endometriosis and find an effective treatment.

Protein ubiquitination, a highly regulated process, is involved in various physiological and pathological mechanisms in cells (Iglesias-Gato et al., 2015). OTUB1 as a deubiquitinating enzyme is a member of the ovarian tumor (OTU) family, which negatively regulates the stability and activity of ubiquitination-promoting proteins (Liu et al., 2019; Zhu et al., 2021). OTUB1 is involved in regulating the DNA damage response and in the development of several cancers (Iglesias-Gato et al., 2015). In breast cancer, OTUB1 promotes the protein stability of MYC through deubiquitination and mediates HK2 expression to increase aerobic glycolysis (Han et al., 2022). In addition, OTUB1 plays an essential regulatory role in the development of lung, esophageal squamous, and colon cancers (Baietti et al., 2016; Zhou et al., 2014, 2018). However, the role of OTUB1 in endometriosis remains unclear.

Heat shock factor 1 (HSF1) is a major transcription factor that promotes the expression of heat shock protein (HSP) in response to endogenous and exogenous stresses (Wang et al., 2020). HSF1 protects cells from stresses such as chemicals, temperature, and radiation and plays a central role in promoting the refolding of misfolded proteins (Dong et al., 2019; Kovács et al., 2019). HSF1 plays a vital role in cancer development and indicates poor cancer prognosis (Fok et al., 2018; Yang et al., 2019). In addition, HSF1 regulates glycolysis through the upregulation of PFKFB3 expression, which ultimately promotes the development of endometriosis (Wang et al., 2021). However, the mechanism controlling the HSF1 protein stability remains unclear.

Here, we identify OTUB1 as a new HSF1-binding protein. OTUB1 promotes the protein stability of HSF1 through deubiquitination. We also find that OTUB1 promotes the growth and invasion of endometriosis cells. Moreover, the development of endometriosis is inhibited in an OTUB1-knockout mouse model. Taken together, our study reveals the theoretical basis for the OTUB1/HSF1 axis as a potential target for the treatment of endometriosis.

## Results

### Ovarian tumor B1 is a novel binding partner for heat shock transcription factor 1

**Figure 1:**
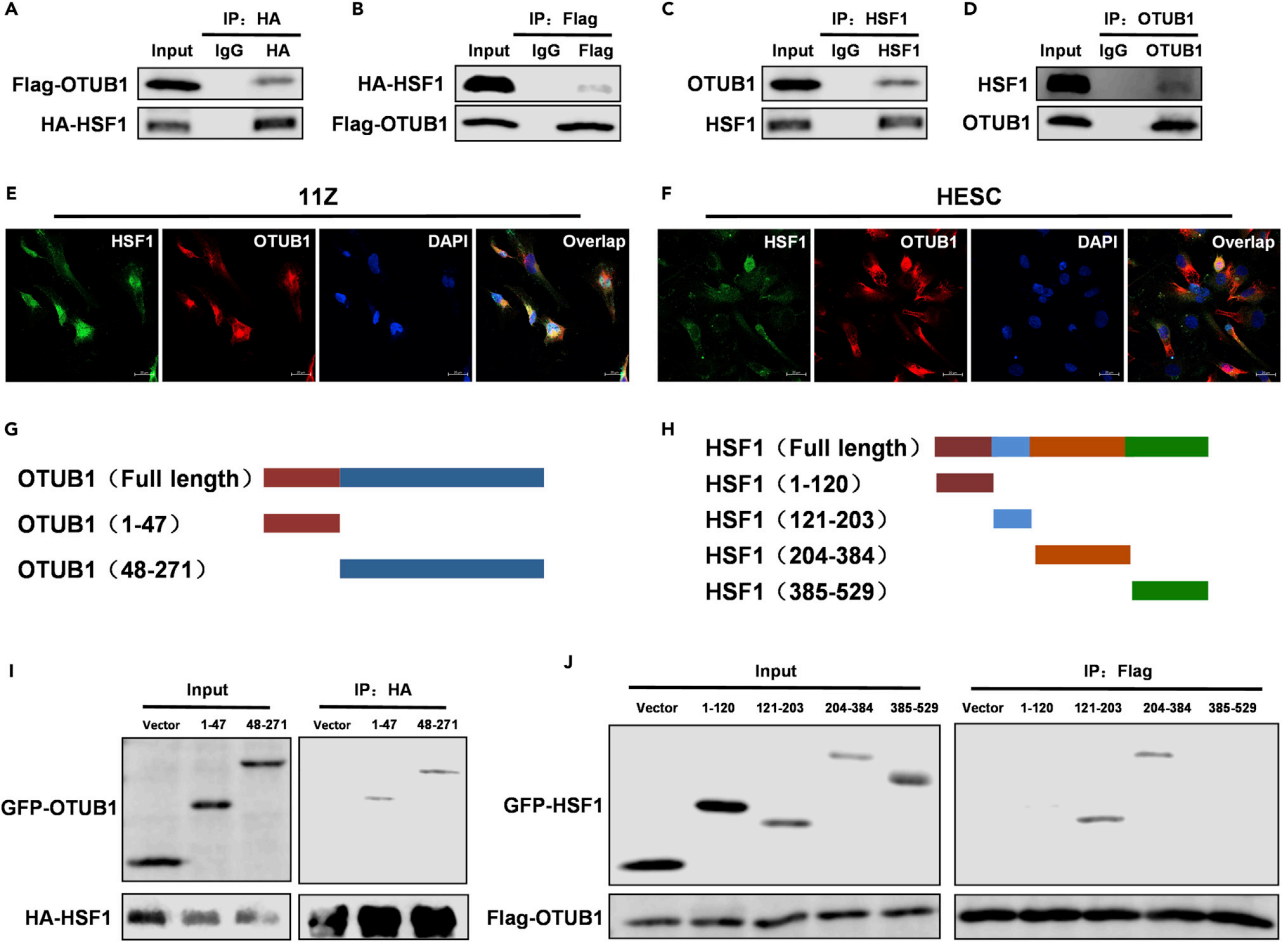
OTUB1 is a novel binding partner for HSF1 (A and B) HA-tagged HSF1 and Flag-tagged OTUB1 were co-transfected with HEK293T cells. Immunoprecipitation was performed with anti-HA agarose or anti-Flag agarose. Immunoprecipitates and cell extracts were subjected to SDS-PAGE analysis and immunoblotting with the indicated antibodies. (C and D) Lysates of 11Z cells were immunoprecipitated with antibodies against HSF1 or OTUB1. Immunoprecipitates and cell extracts were subjected to SDS-PAGE analysis and immunoblotting with the indicated antibodies. (E and F) Confocal immunofluorescence microscopy analyzed the interaction of endogenous OTUB1 and HSF1 proteins in 11Z and HESC cells (scale bar, 20 μm). (G and H) Schematic representation of various OTUB1 and HSF1 truncations. (I) HA-tagged HSF1 and GFP-tagged different OTUB1 truncations were co-transfected into HEK293T cells and immunoprecipitated with anti-HA agarose. Immunoprecipitates and cell extracts were subjected to SDS-PAGE analysis and immunoblotting with the indicated antibodies. (J) Flag-tagged OTUB1 and GFP-tagged different HSF1 truncations were co-transfected into HEK293T cells and immunoprecipitated with anti-Flag agarose. Immunoprecipitates and cell extracts were subjected to SDS-PAGE analysis and immunoblotting with the indicated antibodies.

HSF1 is a vital regulator of the heat shock response and has an essential role in developing endometriosis (Levi-Galibov et al., 2020; Wang et al., 2021). To further elucidate the mechanism that maintains the stability of HSF1 protein, we used HSF1 as bait for mass spectrometry analyses and identified OTUB1 as a novel binding partner of HSF1 (Data S1). To verify this result, we performed a Co-IP analysis. In HEK293T cells, exogenous overexpression of OTUB1 interacted with exogenous overexpression of HSF1 (Figures 1A and 1B). Similarly, there was an interaction between endogenous OTUB1 and HSF1 in the endometrial ectopic epithelial (11Z) cell line (Figures 1C and 1D). In addition, we found that OTUB1 and HSF1 co-localized both in the cytoplasm and nucleus of 11Z and HESC cells (Figures 1E and 1F). Moreover, the OTUB1-HSF1 interaction was demonstrated in the proximity ligation assay (PLA) (Figures S1A and S1B). To determine the regions for interaction between OTUB1 and HSF1, we constructed two truncated fragments of OTUB1 (1-47aa and 48-271aa) (Figure 1G). In the same way, we generated four HSF1 truncations, amino acids 1-120, 121-203, 204-384, and 385-529 (Figure 1H). The results showed that both fragments of OTUB1 could bind to HSF1 (Figure 1I). Both residue 121-203aa and residue 204-384aa of HSF1 were required for interaction with OTUB1 (Figure 1J). Taken together, our results suggest that OTUB1 is a novel binding partner for HSF1.

### Ovarian tumor B1 promotes heat shock transcription factor 1 protein stability via the ubiquitin-proteasome pathway

**Figure 2:**
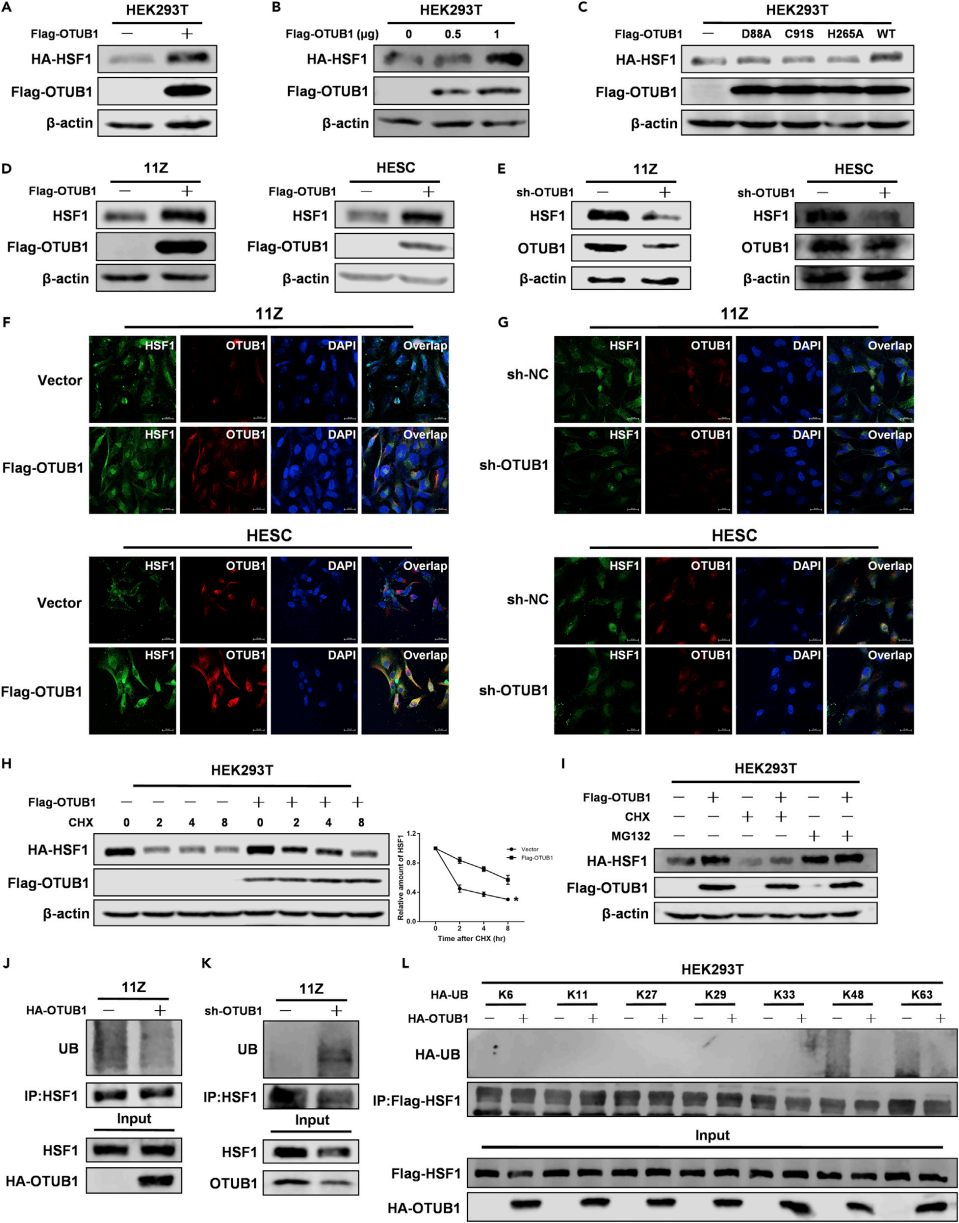
OTUB1 promotes HSF1 protein stability via the ubiquitin-proteasome pathway (A) HA-tagged HSF1 and Flag-tagged OTUB1 were co-transfected with HEK293T cells. (B) HA-tagged HSF1 and Flag-tagged OTUB1 (0, 0.5, 1 μg) were co-transfected with HEK293T cells. (C) HA-tagged HSF1 and Flag-tagged OTUB1 (WT or mutants) were co-transfected with HEK293T cells. (D) Flag-tagged OTUB1 was transfected with 11Z and HESC cells. (E) 11Z and HESC cells were knocked down OTUB1 with shRNA. The above cell extracts were subjected to SDS-PAGE analysis and immunoblotting with the indicated antibodies. (F) Flag-tagged OTUB1 was transfected with 11Z and HESC cells. Confocal immunofluorescence microscopy was performed to analyze the expression of OTUB1 and HSF1 proteins in 11Z and HESC cells (scale bar, 20 μm). (G) 11Z and HESC cells were knocked down OTUB1 with shRNA. Confocal immunofluorescence microscopy was performed to analyze the expression of OTUB1 and HSF1 proteins in 11Z and HESC cells (scale bar, 20 μm). (H) HA-tagged HSF1 and Flag-tagged OTUB1 were co-transfected with HEK293T cells, and the cells were treated with cycloheximide (CHX) for the corresponding time. (I) HA-tagged HSF1 and Flag-tagged OTUB1 were co-transfected with HEK293T cells, and the cells were treated with cycloheximide (CHX) or MG132 (100 μmol/L) for 8 h. The above cell extracts were subjected to SDS-PAGE analysis and immunoblotting with the indicated antibodies. (J and K) HA-tagged OTUB1 was transfected into 11Z cells, or OTUB1 was knocked down in 11Z cells using shRNA. After 48 h, cells were treated with MG132 for 8 h (L) HA-tagged OTUB1, Flag-tagged HSF1 and different ubiquitin mutants were transfected simultaneously with HEK293T cells. The above immunoprecipitates and cell extracts were subjected to SDS-PAGE analysis and immunoblotting with the indicated antibodies.

To further explore the functional consequences of the interaction, we investigated whether OTUB1 could affect the protein level of HSF1. The results showed that OTUB1 overexpression increased the abundance of HSF1 in a dose-dependent manner in HEK293T cells (Figures 2A and 2B). And OTUB1 promoted HSF1 protein stability in dependence on its deubiquitinating enzyme activity (Figure 2C). In addition, overexpression of OTUB1 promoted the abundance of HSF1 in 11Z and HESC cells (Figure 2D). Correspondingly, when OTUB1 was knocked down, the abundance of HSF1 decreased in both cells (Figure 2E). Through immunofluorescence experiments, we obtained the same results (Figures 2F and 2G). We next treated the cells with cycloheximide (CHX) for half-life analysis to verify the role of OTUB1 in regulating HSF1 stability. As we expected, OTUB1 prolonged the half-life of HSF1 in HEK293T cells (Figure 2H). OTUB1 regulates protein stability through the proteasome pathway (Han et al., 2022). Therefore, we overexpressed OTUB1 in HEK293T cells and treated them with cycloheximide or proteasome inhibitors to determine whether the inhibition of the proteasome could abolish OTUB1-mediated stability of HSF1 protein. The results showed that OTUB1 induced-HSF1 protein stability was abolished in the presence of MG132, which suggested that OTUB1 regulated HSF1 protein stability through the ubiquitin-proteasome pathway (Figure 2I). Next, we investigated the effect of OTUB1 on HSF1 ubiquitination. The ubiquitination level of HSF1 was decreased upon overexpression of OTUB1 in 11Z cells (Figure 2J). In contrast, the ubiquitination level of HSF1 was increased when OTUB1 was knocked down in 11Z cells (Figure 2K). And *in vitro* experiment showed that OTUB1 directly deubiquitinated HSF1 (Figure S2A). To determine which lysine (K)-linked polyubiquitin chain HSF1 conjugates with, we overexpressed several ubiquitin mutants. The data showed that HSF1 is preferentially bound to the lysine (K) 48-linked polyubiquitin chain (Figure 2L). In summary, OTUB1 promotes the HSF1 protein stability through the deubiquitination pathway.

### Ovarian tumor B1 enhances heat shock transcription factor 1 transcriptional activity

**Figure 3:**
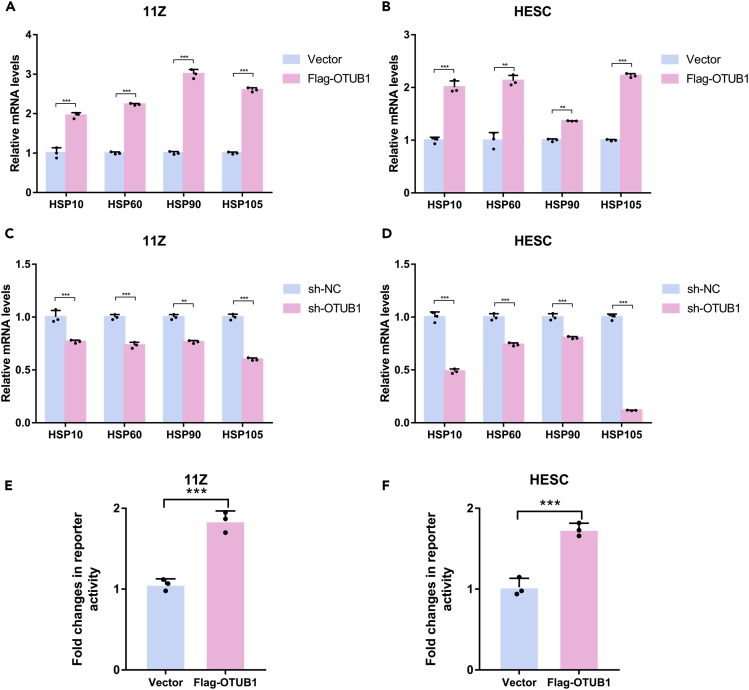
OTUB1 enhances HSF1 transcriptional activity (A and B) Flag-tagged OTUB1 was transfected with 11Z and HESC cells. The mRNA levels were quantitated by RT-PCR. (C and D) 11Z and HESC cells were knocked down OTUB1 with shRNA. The mRNA levels were quantitated by RT-PCR. (E and F) Flag-tagged OTUB1 and dual HSF1 reporter plasmids were transfected with 11Z and HESC cells and detected by luciferase reporter assay. The results of all experiments were expressed as the mean ± SD of three independent experiments, and the Student’s *t* test was used for data analysis. (∗∗p < 0.01, ∗∗∗p < 0.001).

OTUB1 promoted the protein stability of HSF1, so we next determined whether OTUB1 could regulate HSF1-mediated transcription. OTUB1 overexpression increased the mRNA levels of HSF1 target genes, including *HSP10*, *HSP60*, *HSP90*, and *HSP105*, in 11Z and HESC cells (Figures 3A and 3B). And when OTUB1 was knocked down, the mRNA levels of HSF1 target genes were reduced in 11Z and HESC cells (Figures 3C and 3D). In addition, luciferase reporter assay showed that OTUB1 significantly enhanced the activity of human HSP70 promoter in 11Z and HESC cells (Figures 3E and 3F). Thus, OTUB1 enhances the transcriptional activity of HSF1.

### Ovarian tumor B1 promotes endometriosis cell proliferation and migration

**Figure 4:**
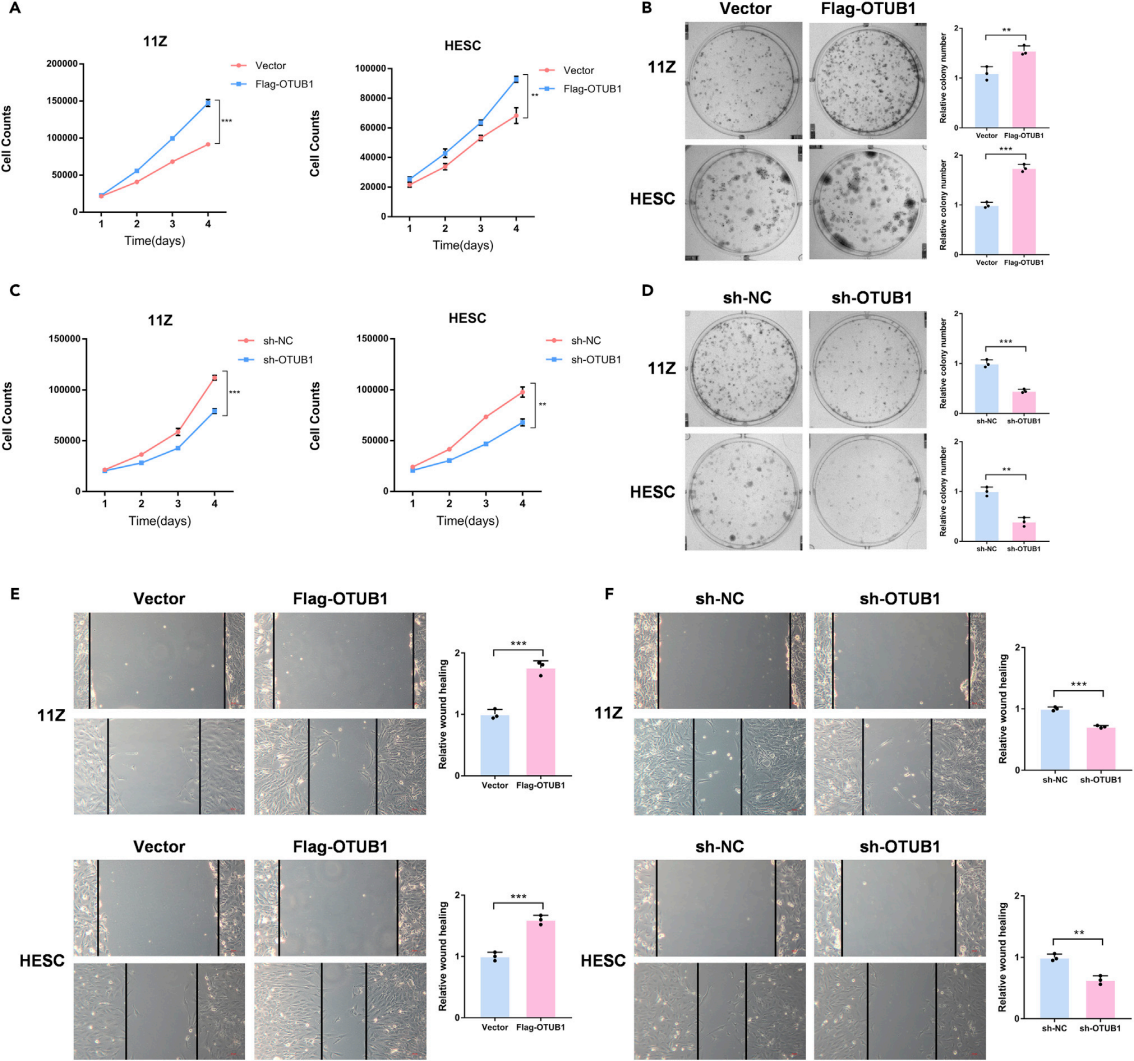
OTUB1 promotes endometriosis cell proliferation and migration (A) Flag-tagged OTUB1 was transfected with 11Z and HESC cells. Cell proliferation analysis was performed. (B) Flag-tagged OTUB1 was transfected with 11Z and HESC cells. Colony formation assay was performed. (C) 11Z and HESC cells were knocked down OTUB1 with shRNA. Cell proliferation analysis was performed. (D) 11Z and HESC cells were knocked down OTUB1 with shRNA. Colony formation assay was performed. (E) Flag-tagged OTUB1 was transfected with 11Z and HESC cells. Wound healing assay was performed (scale bar, 50 μm). (F) 11Z and HESC cells were knocked down OTUB1 with shRNA. Wound healing assay was performed (scale bar, 50 μm). The results of all experiments were expressed as the mean ± SD of three independent experiments, and the Student’s *t* test was used for data analysis (*∗∗*p < 0.01, *∗∗∗*p < 0.001).

To determine the role of OTUB1 in the growth of endometriosis tissue, we examined the effect on endometriosis cell growth when OTUB1 expression was altered. We confirmed the successful overexpression or knockdown of OTUB1 in 11Z and HESC cells at the protein level (Figures S3A and S3B). The results showed that OTUB1 overexpression promoted the proliferation and growth of 11Z and HESC cells (Figures 4A and 4B). In contrast, when OTUB1 was knocked down in 11Z and HESC cells, the growth and proliferation of both cells were inhibited (Figures 4C and 4D). In addition, by wound healing assays, we found that the migration ability of cells was enhanced when OTUB1 was overexpressed and diminished when OTUB1 was knocked down (Figures 4E and 4F). Taken together, OTUB1 promotes the proliferation and migration of endometriosis cells.

### Ovarian tumor B1 promotes epithelial-mesenchymal transition in endometriosis cells

**Figure 5:**
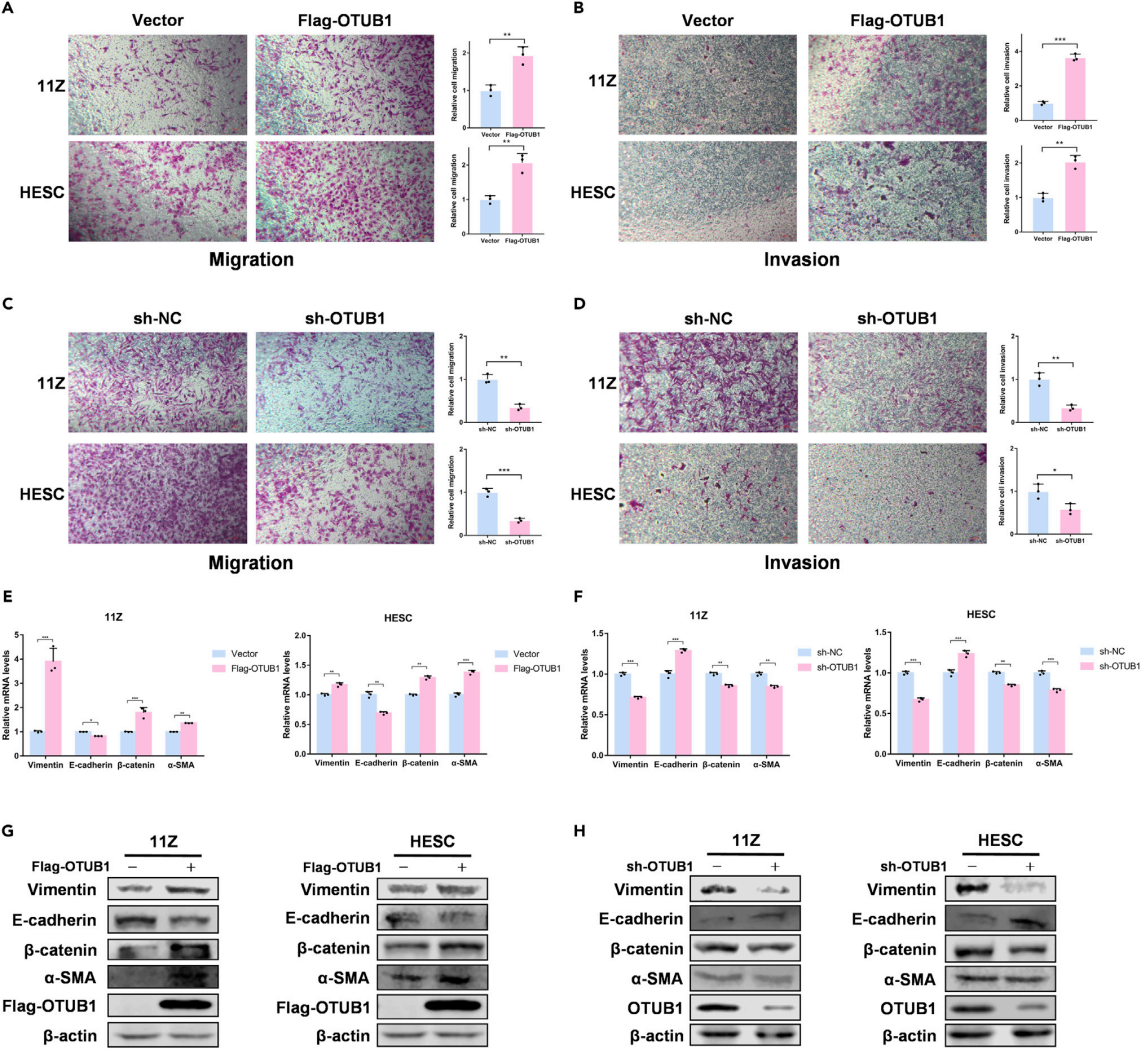
OTUB1 promotes epithelial-mesenchymal transition in endometriosis cells (A and B) Flag-tagged OTUB1 was transfected with 11Z and HESC cells. Transwell migration assay and matrigel invasion assay were performed (scale bar, 50 μm). (C and D) 11Z and HESC cells were knocked down OTUB1 with shRNA. Transwell migration assay and matrigel invasion assay were performed (scale bar, 50 μm). (E) Flag-tagged OTUB1 was transfected with 11Z and HESC cells. The mRNA levels were quantitated by RT-PCR. (F) 11Z and HESC cells were knocked down OTUB1 with shRNA. The mRNA levels were quantitated by RT-PCR. (G) Flag-tagged OTUB1 was transfected with 11Z and HESC cells. Cell extracts were subjected to SDS-PAGE analysis and immunoblotting with the indicated antibodies. (H) 11Z and HESC cells were knocked down OTUB1 with shRNA. Cell extracts were subjected to SDS-PAGE analysis and immunoblotting with the indicated antibodies. The results of all experiments were expressed as the mean ± SD of three independent experiments, and the Student’s *t* test was used for data analysis. (∗p < 0.05, ∗∗p < 0.01, ∗∗∗p < 0.001).

To further explore the effect of OTUB1 on cell migration and invasion ability, we performed transwell migration assays and matrigel invasion assays in 11Z and HESC cells. The results showed that OTUB1 overexpression enhanced the migration and invasion ability of both cells (Figures 5A and 5B). But the results were reversed when OTUB1 was knocked down (Figures 5C and 5D). During epithelial-mesenchymal transition (EMT), the migration and invasive capacity of cells increases (De Craene and Berx, 2013). Also, EMT plays a crucial role in the development of endometriosis (Liu et al., 2020; Weimar et al., 2013). Therefore, we examined mRNA levels of EMT-related factors when OTUB1 was overexpressed in 11Z and HESC cells. The results showed that *E-cadherin* expression was decreased, and the expression of *Vimentin*, *β-catenin* and *α-SMA* were upregulated (Figure 5E). In contrast, the knockdown of OTUB1 in 11Z and HESC cells increased the expression of *E-cadherin* and decreased the expression of *Vimentin*, *β-catenin*, and *α-SMA* at the mRNA level (Figure 5F). We then used Western blot to detect EMT-related factors and the results were consistent with its mRNA levels (Figures 5G and 5H). Together, OTUB1 promotes epithelial-mesenchymal transition in endometriosis cells.

### Ovarian tumor B1 promotes glycolysis in endometriosis cells

**Figure 6:**
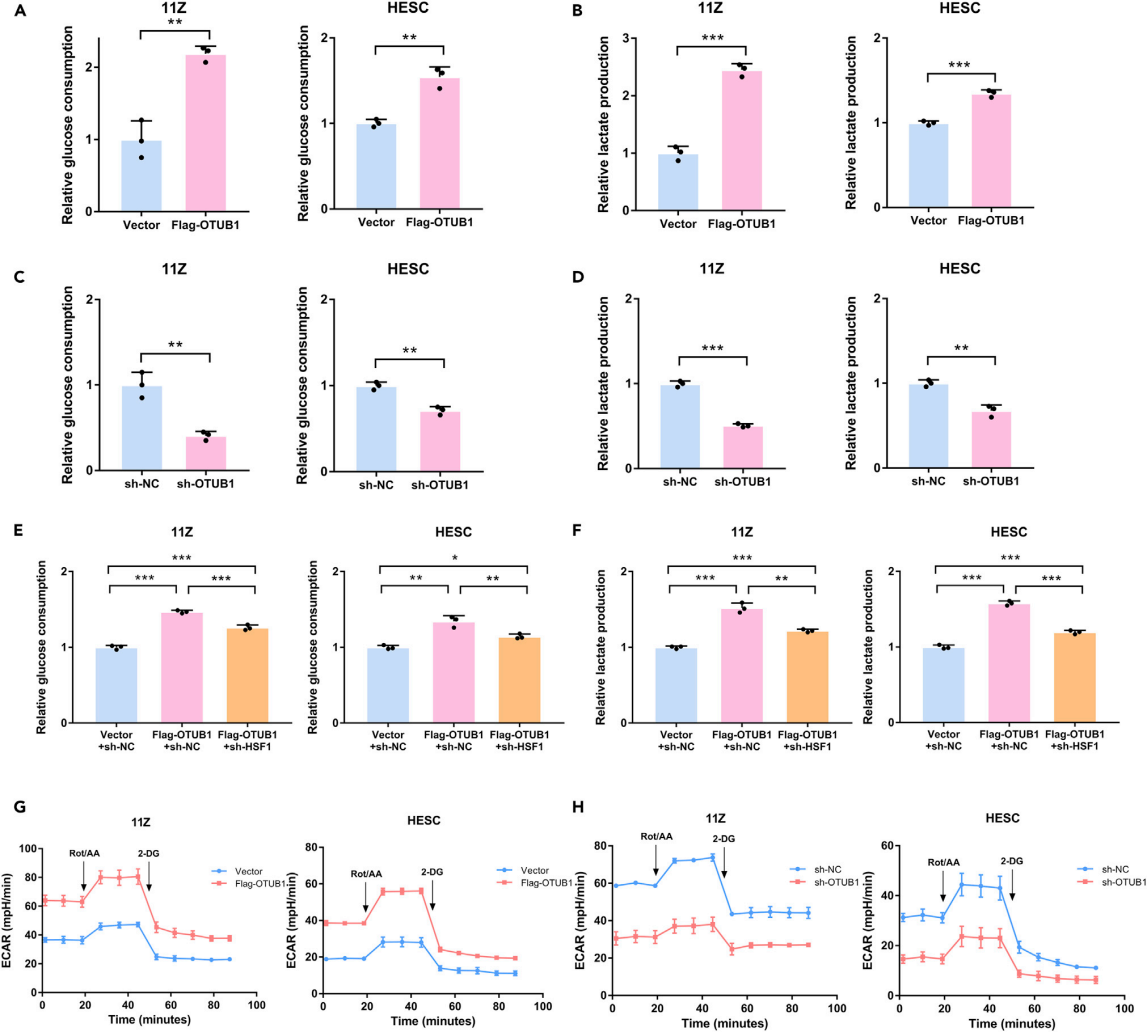
OTUB1 promotes glycolysis in endometriosis cells (A and B) Flag-tagged OTUB1 was transfected with 11Z and HESC cells. (C and D) 11Z and HESC cells were knocked down OTUB1 with shRNA. (E and F) Flag-tagged OTUB1 was transfected with knockdown HSF1 in 11Z and HESC cells. The above glucose consumption and lactate production in the culture medium were detected. (G) Flag-tagged OTUB1 was transfected with 11Z and HESC cells. Glycolytic rate was determined with Seahorse XFe24 Analyzer. (H) 11Z and HESC cells were knocked down OTUB1 with shRNA. Glycolytic rate was determined with Seahorse XFe24 Analyzer. The results of all experiments were expressed as the mean ± SD of three independent experiments, and the Student’s *t* test was used for data analysis. (∗∗p < 0.01, ∗∗∗p < 0.001).

Endometriosis lesions use aerobic glycolysis to produce energy (Young et al., 2016). To verify the effect of OTUB1 on glycolysis, we examined the glucose and lactate levels in the culture medium after OTUB1 overexpression or knockdown. The results showed that overexpression of OTUB1 increased glucose consumption and lactate production in 11Z and HESC cells (Figures 6A and 6B). In contrast, glucose consumption and lactate production were reduced when OTUB1 was knocked down in both cells (Figures 6C and 6D). Furthermore, our data suggested that HSF1 was required for OTUB1-mediated aerobic glycolysis (Figures 6E and 6F). The regulation of glycolysis by HSF1 is dependent on PFKFB3, a key enzyme of glycolysis (Wang et al., 2021). Therefore, we examined the expression of key enzymes of glycolysis (PFKFB3, PKM2, HK2) when OTUB1 was overexpressed or knocked down. The results showed that OTUB1 promoted glycolysis via HSF1-mediated PFKFB3 expression (Figures S4A and S4B). We next measured the glycolytic rate of 11Z and HESC cells using the Seahorse Bioscience Flux Analyzer. The results showed that the rate of glycolysis was enhanced in both cells when OTUB1 was overexpressed (Figure 6G). And the opposite result was obtained when OTUB1 was knocked down (Figure 6H). In conclusion, OTUB1 promotes glycolysis in endometriosis cells.

### Ovarian tumor B1 promotes endometriosis growth *in vivo*

**Figure 7:**
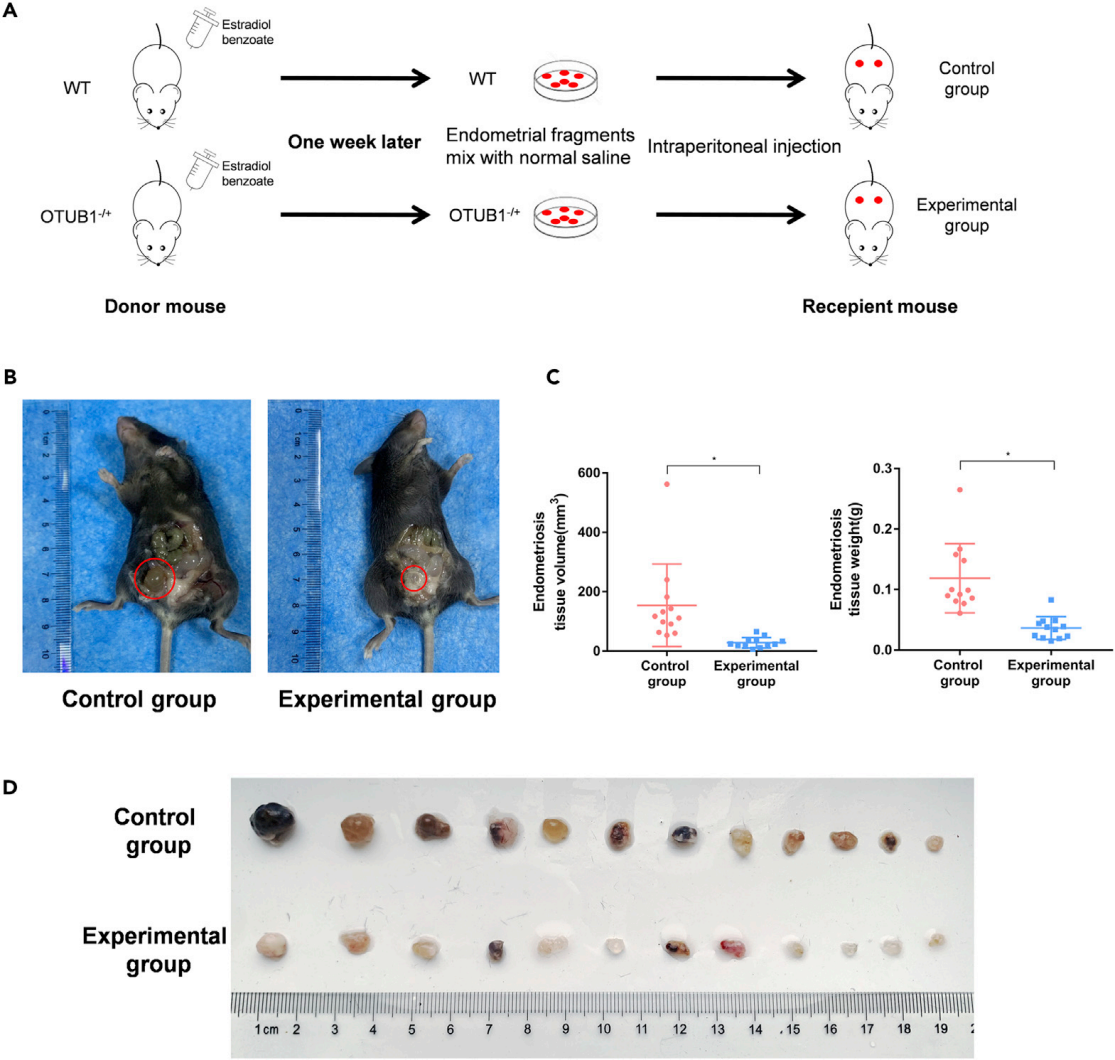
OTUB1 promotes endometriosis growth *in vivo* (A) A mouse model of endometriosis in OTUB1-knockout mice was established. (B–D) Endometriotic lesions were removed from the experimental (n = 12) and control groups (n = 12) and the volume and weight were measured. The results of all experiments were expressed as the mean ± SD, and the Student’s *t* test was used for data analysis. (∗p < 0.05).

OTUB1 homozygote mice die at the embryonic stage, but OTUB1 heterozygous mice also exhibit phenotypes such as reduced grip strength and body weight (Pasupala et al., 2018; Ruiz-Serrano et al., 2021; Saldana et al., 2019). We, therefore, established an endometriosis model using OTUB1-knockout mice to investigate the effect of OTUB1 on the development of endometriosis *in vivo* (Figure 7A). The results showed that the volume of endometriosis tissues in the experimental group of mice was significantly smaller than that in the control group (Figure 7B). We next counted the weight and volume of all endometriosis tissues in the experimental and control groups. The data indicated that the growth of endometriotic tissues was inhibited in the experimental group (Figures 7C and 7D). Thus, the knockdown of OTUB1 inhibits the development of endometriosis *in vivo*.

### Ovarian tumor B1 expression is positively correlated with heat shock transcription factor 1 in endometriosis tissues

**Figure 8:**
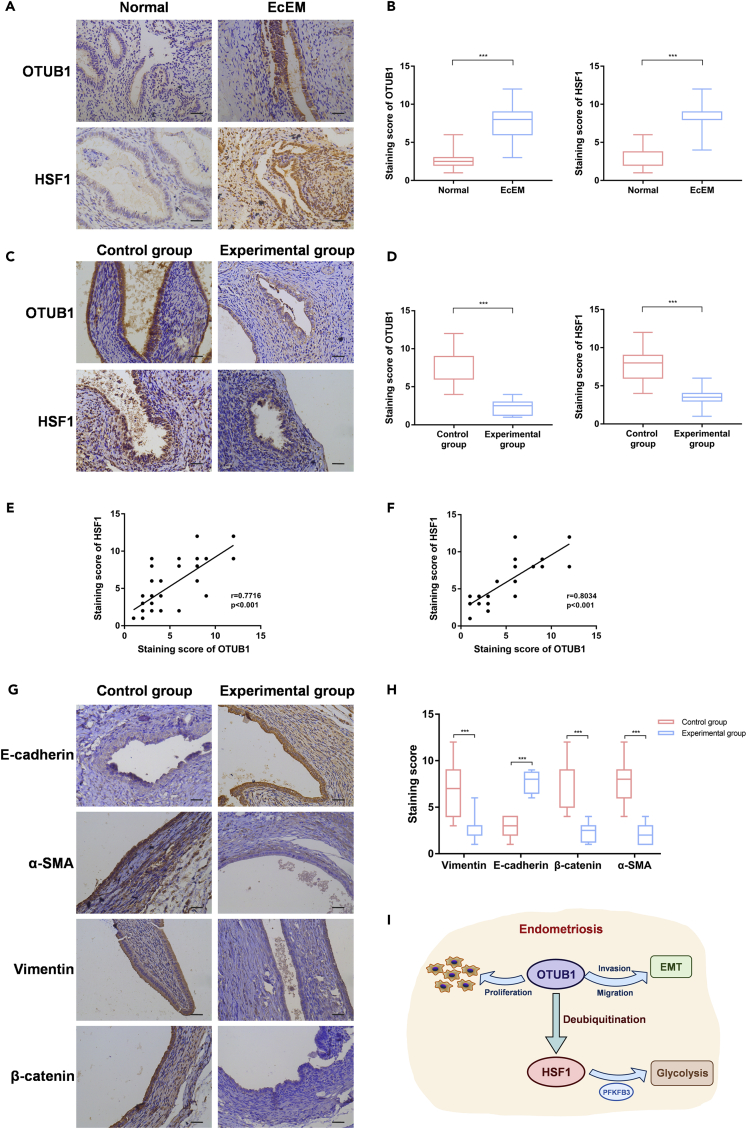
OTUB1 expression is positively correlated with HSF1 in endometriosis tissues (A and B) Immunohistochemical for OTUB1 and HSF1 expression in human normal (n = 20) and ectopic endometrial tissues (n = 20) with semi-quantitative staining analysis (scale bar, 20 μm). (C and D) Immunohistochemical for OTUB1 and HSF1 expression in endometriotic tissues of the experimental (n = 12) and control groups (n = 12) in the above animal experiments with semi-quantitative staining (scale bar, 20 μm). (E and F) Pearson correlation analysis was performed on OTUB1 and HSF1 semi-quantitative staining scores. (G and H) Immunohistochemical for EMT-related factors expression in endometriotic tissues of the experimental (n = 12) and control groups (n = 12) in the above animal experiments with semi-quantitative staining analysis (scale bar, 20 μm). (I) Schematic model of mechanism that OTUB1-mediated HSF1 deubiquitination promoted glycolysis and development of endometriosis. The results of all experiments were expressed as the mean ± SD, and the Student’s *t* test was used for data analysis. (*∗∗∗*p < 0.001).

To further explore the correlation between the expression of OTUB1 and HSF1 in endometriotic tissues, we analyzed the expression of OTUB1 and HSF1 in normal endometrium and endometriotic tissues by IHC assays. IHC staining showed high expression of OTUB1 and HSF1 in endometriotic tissues (Figures 8A and 8B). We next examined the expression of OTUB1 and HSF1 by IHC assays in the tissues obtained from the above animal experiments. The results showed that both OTUB1 and HSF1 expression were reduced in the ectopic tissues of the experimental group (Figures 8C and 8D). And the expression of OTUB1 and HSF1 were positively correlated in endometriotic tissues (Figure 8E). We obtained the same results in the endometriotic tissues of mice (Figure 8F). Similarly, we examined the expression of EMT-related factors in endometriotic tissues of mice. The results confirmed that OTUB1 promoted EMT in endometriosis, which is consistent with our previous findings (Figures 8G and 8H). In conclusion, the OTUB1 and HFS1 expressions in endometriotic tissues are positively correlated.

## Discussion

Endometriosis is a benign disease but has a malignant biological behavior similar to cancer, such as migration and invasion (Liu et al., 2017). Deubiquitinating enzymes (DUBs) remove ubiquitin chains from post-translationally modified proteins to stabilize them and play a crucial role in tumorigenesis and metastasis (He et al., 2017; Komander et al., 2009). OTUB1 acts as a deubiquitinating enzyme that regulates ubiquitination and promotes the stabilization of tumorigenesis-associated proteins to participate in tumor progression (Liao et al., 2022). OTUB1 mediates the deubiquitination of FOXM1 to promote renal cancer tumor progression (Zhou et al., 2020). In addition, OTUB1 plays a vital role in regulating the stability of SLC7A11 and the CD44-mediated effects on ferroptosis in tumors (Liu et al., 2019). HSF1 is a transcription factor that promotes tumorigenesis by regulating cancer-specific transcription (Mendillo et al., 2012). Moreover, HSF1 plays an essential role in developing endometriosis (Wang et al., 2021). However, the upstream mechanisms regulating this process remain unclear.

In this study, we identified the interaction between OTUB1 and HSF1. OTUB1 promoted the protein stability of HSF1 through deubiquitination and prolonged its half-life. In addition, OTUB1 promoted the transcriptional activity of HSF1. Thus, OTUB1 is a novel regulator of HSF1. Moreover, OTUB1 plays a vital role in the pathogenesis and aggressive biology of cancers (Weng et al., 2016). The expression level of OTUB1 correlates with tumor size and differentiation and predicts poor prognosis (Liu et al., 2014). In this study, OTUB1 enhanced the proliferation, migration, and invasion of endometriosis cells. EMT manifested by an increased capacity for cell migration and invasion is thought to be a prerequisite for establishing endometriotic lesions (De Craene and Berx, 2013; Yang and Yang, 2017). Our data showed that overexpression of OTUB1 altered the expression levels of EMT-related factors and promotes EMT in endometriosis cells. And OTUB1 promoted glycolysis in endometriosis cells. In addition, OTUB1-knockout mice show late embryonic death, confirming that OTUB1 plays a crucial role in embryonic development (Pasupala et al., 2018). We established an endometriosis model using OTUB1-knockout mice. The results showed that OTUB1 deletion inhibited the growth of endometriosis lesions.

In summary, our study identifies a HSF1 new binding partner-OTUB1, which promotes its protein stability via deubiquitination. The results further show that OTUB1 promotes the development of endometriosis *in vivo* and *in vitro* (Figure 8I). Therefore, OTUB1 may serve as a novel biomarker and provide new ideas and approaches for the treatment of endometriosis.

### Limitations of the study

In this study, we demonstrate that OTUB1 promotes the development of endometriosis via deubiquitination of HSF1. The function of OTUB1 as a deubiquitinating enzyme acting on other substrates in endometriosis is still unknown. In addition, more work is needed to explore other roles of OTUB1 in the mechanism of endometriosis development.

## STAR★Methods

### Key resources table

**Table:** 

REAGENT or RESOURCE	SOURCE	IDENTIFIER
**Antibodies**		

Mouse anti-OTUB1	Santa Cruz	Cat#sc-130458; RRID: AB_2236433
Rabbit anti-OTUB1	Abcam	Cat#ab175200
Mouse anti-HSF1	Santa Cruz	Cat#sc-17757; RRID: AB_627753
Rabbit anti-HSF1	Abcam	Cat#ab52757; RRID: AB_880518
Rabbit anti-Ubiquitin	Proteintech	Cat#10201-2-AP; RRID: AB_671515
Mouse anti-Vimentin	Proteintech	Cat#60330; RRID: AB_2881439
Rabbit anti-E-cadherin	Proteintech	Cat#20874-1-AP; RRID: AB_10697811
Rabbit anti-α-SMA	Proteintech	Cat#14395-1-AP; RRID: AB_2223009
Rabbit anti-β-catenin	Proteintech	Cat#51067-2-AP; RRID: AB_2086128

**Chemicals, peptides, and recombinant proteins**		

Cycloheximide	MedChemExpress	Cat#HY-12320
MG132	MedChemExpress	Cat#HY-13259
Lactate assay kit	Biovision	Cat #k627-100
Glucose (GO) assay kit	Sigma	Cat #GAGO20-1KT

**Experimental models: Cell lines**		

HEK293T cells	Cell Bank of the Chinese Academy of Sciences	Cat#GNHu17
11Z cells	Zeitvogel et al. (2001)	N/A
HESC cells	Krikun et al. (2004)	N/A

**Experimental models: Organisms/strains**		

OTUB1 KO mice	Cyagen Biosciences	S-KO-00499

**Oligonucleotides**		

Primer for construct plasmids and RT-PCR, see Table S2	This paper	N/A

**Software and algorithms**		

Prism 7.0	GraphPad Software	N/A

### Resource availability

#### Lead contact

Further information and requests for resources and reagents should be directed to and will be fulfilled by the Lead Contact, Zhenhai Yu at tomsyu@163.com.

#### Materials availability

This study did not generate new unique reagents.

### Experimental model and subject details

#### Mice

The animal experiments described in this study were approved by the Ethics Committee of Weifang Medical University (2022SDL220). All mice used in this study were female between 5 and 9 weeks. OTUB1-knockout mice were purchased from Cyagen Biosciences. All animals were housed under pathogen-free conditions, and performed according to the National Institutes of Health’s Guide for the Care and Use of Laboratory Animals.

#### Cell lines

The endometrial ectopic epithelial (11Z) cell line and the endometrial stromal (HESC) cell line were constructed by Anna Starzinski-Powitz and Graciela Krikun respectively (Krikun et al., 2004; Zeitvogel et al., 2001). The HEK293T cell line was from the Chinese Academy of Sciences. All cell lines were cultured in a 37°C incubator containing 5% CO_2_ using DMEM or DMEM/F-12 medium (CORNING) containing 10% FBS (CORNING) and penicillin-streptomycin solution (100 μg/mL penicillin and 100 μg/mL streptomycin).

#### Tissue samples

There were 20 tissue samples from each of the experimental and control groups in this study. The patients with laparoscopically and follow-up histologically confirmed endometriosis attending the Affiliated Hospital of Weifang Medical College were selected as the experimental group. Tissue samples for the experimental group were obtained from ectopic tissues of patients with endometriosis. Tissue samples for the control group were obtained from normal endometrium of patients with no endometriosis or no history of endometriosis. All patients had regular menstrual cycles and had not received hormone therapy in the past six months. The characteristics of all recruited subjects are listed in Supplementary material (Table S2). This study was approved by the Ethics Committee of Affiliated Hospital of Weifang Medical University (wyfy-2022-ky-099).

### Method details

#### Immunohistochemistry (IHC)

Normal endometrium or ectopic tissues were fixed with paraformaldehyde and sectioned. Sections were subjected to antigen repair, closed using H_2_O_2_, and incubated with primary antibodies. Sections were then incubated in HRP-conjugated goat anti-rabbit or mouse IgG incubation. Then, sections were stained using DAB chromogen substrate solution and hematoxylin. Immunohistochemically stained tissues were scored by the percentage of positive cells and intensity of staining. The percentage of positive cells was graded as 0 (0–5%), 1 (6–25%), 2 (26–50%), 3 (51–75%), and 4 (76–100%), and the staining intensity was graded as 0 (no staining), 1 (weak staining), 2 (moderate staining), and 3 (strong staining). The semi-quantitative score ranged from 0 to 12, and a score greater than 4 was considered positive.

#### Immunofluorescent analysis and proximity ligation assay (PLA)

The indicated cells were seeded in 24-well plates at 80,000–100000 cells per well. 24 h later, cells were fixed using 4% paraformaldehyde and then treated with 0.2% Triton X-100. Cells were blocked using BSA and incubated overnight at 4°C with primary antibodies. The next day cells were incubated with fluorescent-conjugated secondary antibodies and then added with mounting medium with DAPI. PLA was performed using the Duolink *In Situ* Red assay (Sigma, DUO92101) following manufacturer’s instructions (Ren et al., 2022). Cells were incubated with PLUS and MINUS PLA probes for 1 h at 37°C. Then cells were incubated in ligation solution for 30 min at 37°C and in amplification solution for 100 min at 37°C. And cells were observed after mounting with DAPI. The results were visualized by a confocal microscope (ZEISS).

#### Immunoprecipitation and western blot analysis

The indicated cells were lysed in lysis buffer containing protease inhibitors (Sigma–Aldrich), followed by incubation with the indicated beads and antibodies overnight at 4°C (Lu et al., 2020). For western blotting, the indicated cells were collected and then lysed on ice using lysis buffer (Beyotime, P0013). Then the supernatant was mixed with 5×loading buffer and placed at 100°C for 10 min. The protein samples were separated using SDS-PAGE, transferred to PVDF membranes, and incubated with the indicated antibodies. The proteins were visualized by odyssey instrument. The information of plasmids and antibodies was provided in Supplementary material (Table S1).

#### *In vitro* deubiquitination assay

HA-tagged ubiquitin and Flag-tagged HSF1 were co-transfected with HEK293T cells. The IP assay was performed to get ubiquitinated Flag-tagged HSF1. The HSF1 proteins were incubated with His-OTUB1 using deubiquitinating buffer (5mM MgCl_2_, 60mM HEPES, 4% glycerol, pH 7.5) at 30°C for 4 h. The deubiquitinated proteins were tested by western blot analysis.

#### Real-time PCR and dual-luciferase assay

Total RNA was extracted from indicated cells using the Trizol kit (Omega) and reversed transcription using the cDNA synthesis kit (Takara) to obtain cDNA. Quantitative real-time PCR was then performed using SYBR Green PCR Master Mix (Takara) and CFX96 Real-Time PCR detection system (Bio-Rad). The information on primer sequences was provided in Supplementary material (Table S1). The indicated plasmids, HSF-luc, and pRL-TK were co-transfected with cells. Luciferase assays were performed using dual-luciferases reporter assay kit (Promega) according to manufacturer’s instructions.

#### Cell proliferation analysis, colony formation assay and wound healing assay

##### Cell proliferation analysis

The indicated cells were seeded into 24-well plates at 20,000 cells per well. Cells were counted every 24 h for 4 days.

##### Colony formation assay

The indicated cells were seeded into 6-well plates at 200–1000 cells per well. After 10–14 days of cell culture, the cells were fixed with 4% paraformaldehyde for 15 min and then stained with crystal violet for 20 min. Cells were photographed after drying.

##### Wound healing assay

The indicated cells were seeded into 6-well plates and cultured until a monolayer was formed. The cells were then scribed with a medium-sized pipette tip, washed with PBS, and photographed. 24 h later, the cells were photographed.

#### Transwell migration assay and matrigel invasion assay

The indicated cells were seeded in a membrane supracavity with a pore diameter of 8 μm at 80,000–100000 cells per well. The liquid in the upper chamber was 200μL of DMEM/F12 medium (CORNING), and the liquid in the basement chamber was 600μL of DMEM/F12 medium containing 10% FBS. The upper chamber fluid was mixed with BD Biocoat Matrigel (BD Biosciences) with DMEM/F12 medium (1:8) for matrigel invasion assays. After 12 or 24 h, the chambers were fixed with 4% paraformaldehyde for 60 min, stained with crystalline violet for 30 min, and then photographed.

#### Glucose consumption, lactate production and glycolytic rate

The indicated cells were seeded in six-well plates, and the cultures were collected after 24 h. Glucose and lactate concentrations in the culture medium were determined using the Glucose and Lactate Assay Kit (Sigma, #GAGO20-1kT and Biovision, #K627-100) according to manufacturer’s instructions (Lu et al., 2021, 2022; Yang et al., 2018). The glycolytic rate was determined with Seahorse XFe24 Analyzer (Agilent) (Han et al., 2022). The indicated cells were seeded into XF24 cell culture plates (Seahorse Bioscience) at 20,000 cells per well. The cells were placed in a CO_2_ free incubator at 37°C to equilibrated with bicarbonate-free buffered DMEM for 60 min before the XF analysis. The extracellular acidification rate (ECAR) was analyzed over time after sequential injection of rotenone/antimycin A (Rot/AA) and 2-deoxyglucose (2-DG). After finishing recording, ECAR values were calculated after normalizing with total protein amounts.

#### Generation of OTUB1-knockout mice and establishment of the endometriosis mouse model

OTUB1-knockout (OTUB1^−/+^) mice were generated using CRISPR/Cas9-mediated genome editing in C57BL/6J embryonic stem cell (gRNA1: ACCTTGAAAGTACGCGCAGCTGG; gRNA2: TGGGGACCCATCCTCGGGAAAGG; gRNA3: GTGGAATGTGGAAGCGCGCGTGG; gRNA4: AGAGCAGGGAAGCGCACACTCGG). The donor mice in the experimental group were OTUB1-knockout (OTUB1^−/+^) female mice, and in the control group were C57BL/6J wild-type (WT) mice, with 6 mice in each group. All donor mice were at 5 weeks of age. Donor mice were injected intramuscularly with estradiol benzoate in the thighs at two-day intervals. One week later, the donor mice were executed, and the uterus was removed and cut into 1 mm^3^ size pieces. Each donor uterine fragments were injected equally into the intraperitoneal cavity of both recipient mice. Three weeks later, the mice were executed, and the ectopic tissue in the abdominal cavity were observed and recorded.

### Quantification and statistical analysis

Statistical analysis of all data was performed using Graphpad Prism 7.0 software with a mean ± structural equation using a two-tailed unpaired t-test. Pearson correlation analysis was used to evaluate the relationship between the two variables. p*-values* < 0.05 were considered statistically significant. ∗p < 0.05, ∗∗p < 0.01, ∗∗∗p < 0.001.

## Data Availability

• The raw data reported in this paper will be shared by the lead contact upon request. • This paper does not report original code. • Any additional information required to reanalyze the data reported in this paper is available from the lead contact upon request.

## Supplemental information

Document S1. Figures S1–S4 and Tables S1 and S2

Data S1. (A) HSF1 bonds to protein, related to Figure 1. (B) HSF1 bonds to peptide, related to Figure 1

Data S2. Full images of western blots, related to Figures 1, 2, 5 and S2–S4
