# Supplementary material for: Ovarian tumorB1-mediated heat shock transcription factor 1 deubiquitination is critical for glycolysis and development of endometriosis
Source: iScience. 2022 Oct 14;25(11):105363. doi: 10.1016/j.isci.2022.105363 (PMC9626688; doi:10.1016/j.isci.2022.105363)
Supplement: Document S1. Figures S1–S4 and Tables S1 and S2 [file mmc1.pdf]

iScience, Volume *25*

Supplemental information

Ovarian tumorB1-mediated heat shock transcription factor 1 deubiquitination is critical for glycolysis and development of endometriosis

Xi Ling, Jiayi Lu, Xiaoyun Wang, Lan Liu, Lu Liu, Yadi Wang, Yujun Sun, Chune Ren, Chao Lu, and Zhenhai Yu

Figure S1. The OTUB1-HSF1 interaction was demonstrated in the proximity ligation assay (PLA), related to Figure 1.

(A) Representative images of *in situ* PLA showing the interaction between OTUB1 and HSF1 in 11Z cells (scale bar, 20μm). (B) Representative images of *in situ* PLA showing the interaction between OTUB1 and HSF1 in HESC cells (scale bar, 20μm).

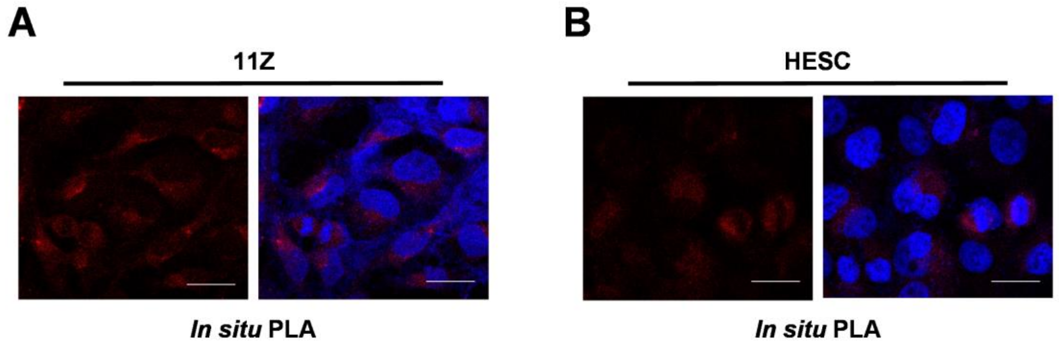

Figure S2. OTUB1 directly deubiquitinated HSF1 in vitro experiment, related to Figure 2.

(A) HA-tagged ubiquitin and Flag-tagged HSF1 was co-transfected with HEK293T cells. HSF1 proteins were purified by IP with Flag beads and elution with Flag peptide. OTUB1 proteins were prified from E. coli. His-OTUB1 and Flag-HSF1 were added into a reaction mixture at 30°C for 4hr. Immunoblotting analyse was performed.

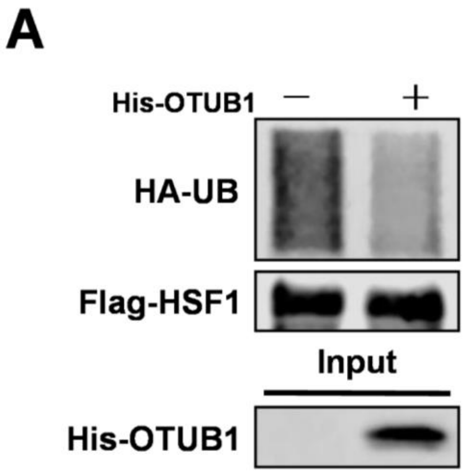

Figure S3. Successful overexpression or knockdown of OTUB1 in 11Z and HESC cells at the protein level, related to Figure 4.

(A) Successful overexpression of OTUB1 was validated by Western blot in 11Z and HESC cells. (B) Successful knockdown of OTUB1 was validated by Western blot in 11Z and HESC cells.

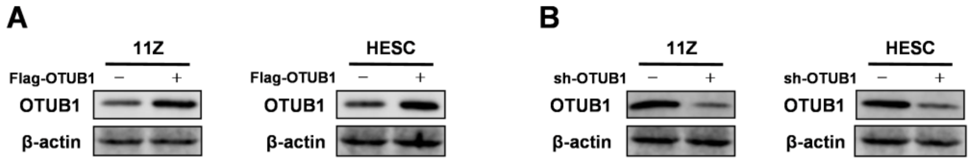

Figure S4. OTUB1 promoted glycolysis via HSF1 mediated PFKFB3 expression, related to Figure 6.

(A) Flag-tagged OTUB1 was transfected with 11Z and HESC cells. (B) 11Z and HESC cells were knocked down OTUB1 with shRNA. The above cell extracts were subjected to SDS-PAGE analysis and immunoblotting with the indicated antibodies.

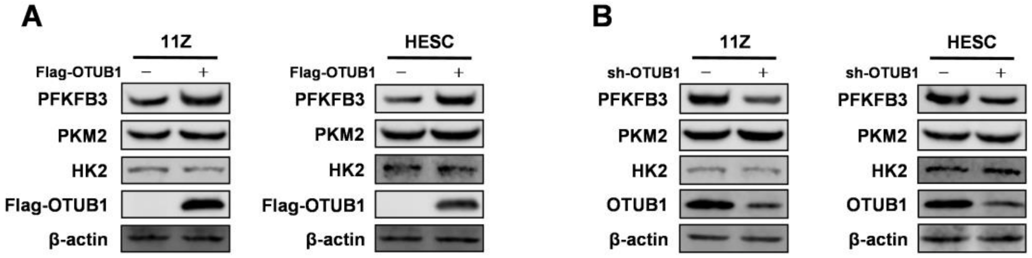

Table S1. Primary antibodies, reagents, shRNA sequences, and primers, related to STAR★Methods.

Primary antibodies and reagents used in this study.

REAGENT or RESOURCE	SOURCE	IDENTIFIER
**Antibodies**		
Mouse anti-HA	Sigma-Aldrich	Cat#H3663
Mouse anti-Flag	Sigma-Aldrich	Cat#F1804
Mouse anti-β-actin	Sigma-Aldrich	Cat#A1978
Rabbit anti-HA	Proteintech	Cat#51064-2-AP
Rabbit anti-Flag	Proteintech	Cat#20543-1-AP
Rabbit anti-β-actin	Proteintech	Cat#20536-1-AP
Mouse anti-OTUB1	Santa Cruz	Cat#sc-130458
Rabbit anti-OTUB1	Abcam	Cat#ab175200
Mouse anti-HSF1	Santa Cruz	Cat#sc-17757
Rabbit anti-HSF1	Abcam	Cat#ab52757
Mouse anti-GFP	Sigma-Aldrich	Cat#G6539
Rabbit anti-GFP	Proteintech	Cat#50430-2-AP
Rabbit anti-Ubiquitin	Proteintech	Cat#10201-2-AP
Mouse anti-Vimentin	Proteintech	Cat#60330
Rabbit anti-E-cadherin	Proteintech	Cat#20874-1-AP
Rabbit anti-α-SMA	Proteintech	Cat#14395-1-AP
Rabbit anti-β-catenin	Proteintech	Cat#51067-2-AP
IRDye 800CW goat anti-rabbit	LI-COR	Cat#925-32210
IRDye 680LT goat anti-mouse	LI-COR	Cat#925-68020
Anti-HA Affinity Gel	Sigma-Aldrich	Cat#E6779
Anti-FLAG Affinity Gel	Sigma-Aldrich	Cat#F2426
Cycloheximide	MedChemExpress	Cat#HY-12320
MG132	MedChemExpress	Cat#HY-13259
Lipofectamine 2000	Thermo Fisher	Cat#11668019
Lactate assay kit	Biovision	Cat #k627-100
Glucose (GO) assay kit	Sigma	Cat #GAGO20-1KT
Bacterial Strain		
E. coli DH5α	Thermo Fisher	Cat#18258012
E. coli Stable 3	Thermo Fisher	Cat#C737303
Experimental Models: Cell Lines		
Human: 293T cells	Cell Bank of the Chinese Academy	Cat#GNHu17
Recombinant DNA		
pCDNA3.0/neo-HA-OTUB1	This paper	N/A

pCDNA3.1/neo-Flag-OTUB1	This paper	N/A
pCDNA3.1/neo-Flag-OTUB1	This paper	N/A
pCDNA3.0/neo-HA-HSF1	This paper	N/A
pCDNA3.1/neo-Flag-HSF1	This paper	N/A
pEGFP-C1-OTUB1 (OT1 or OT2)	This paper	N/A
pEGFP-C1-HSF1 (M1, M2, M3 or	This paper	N/A
pLVX-shRNA1-OTUB1	This paper	N/A
pLVX-shRNA1-HSF1	This paper	N/A
pCDEF-HA-Ub-K6	This paper	N/A
pCDEF-HA-Ub-K11	This paper	N/A
pCDEF-HA-Ub-K27	This paper	N/A
pCDEF-HA-Ub-K29	This paper	N/A
pCDEF-HA-Ub-K33	Addgene	Cat#52961
pRK5-HA-Ub-K48	Addgene	Cat#17605
pRK5-HA-Ub-K63	Addgene	Cat#17606

shRNA sequences used in this study.

shRNA	Sense (5'-3')	Anti-sense (5'-3')
shRNA-Control	TTCTCCGAACGGTCACGT	ACGTGACCGTTCGGAGAA
shRNA-OTUB1	AGGAGTATGCTGAAGATGACA	TGTCATCTTCAGCATACTCCT
shRNA-HSF1	GATCCGCAGGTTGTTCATAGT<br>CAGAATTCAAGAGATTCTGAC<br>TATGAACAACCTGCTTTTTTG	AATTCAAAAAAGCAGGTTGTTC<br>ATAGTCAGAATCTCTTGAATTC<br>TGACTATGAACAACCTGCG

The primers used for real-time PCR in this study.

Gene	Sense (5'-3')	Anti-sense (5'-3')
HSP10	GGAGTGCTGCTGAAACTGTA	TTAGAACCCGATCCAACAGC
HSP60	ACGGCTTGCAAAACTTTCAG	TTAAGGGCATCTGTAACTCTGTC
HSP90	TTCCCTGTAGTTGACAATTC	CTACATTTCCATCCACAAGA
HSP105	CACCAGAAAACCCAGACACT	GGGAGACTGTGAGGTTTGTT
E-cadherin	GACAACAAGCCCGAATT	GGAAACTCTCTCGGTCCA
Vimentin	GAGAACTTTGCCGTTGAAGC	GCTTCCTGTAGGTGGCAATC
α-SMA	AGACATCAGGGGGTGATGGT	CATGGCTGGGACATTGAAAG
β-catenin	ATGGCTACTCAAGCTGATTT	TCCCACTCATACAGGACTTG
18S	GTTGAACCCCATTCGTGATG	GCCTCACTAAACCATCCAA

Table S2. The characteristics of the recruited subjects, related to STAR★Methods.

	Controls	Endometriosis
No of case	20	20
Age (mean ± SD)	32.7 ± 4.1	31.0 ± 5.1
Menstrual cycle phase		
Proliferative	13	12
Secretory	7	8
rASRM stag		
III	-	9
IV	-	11
Type		
Peritoneal endometriosis	-	7
Ovarian endometriosis	-	13
