# Supplementary figures and images for: Ovarian tumorB1-mediated heat shock transcription factor 1 deubiquitination is critical for glycolysis and development of endometriosis
Source: iScience. 2022 Oct 14;25(11):105363. doi: 10.1016/j.isci.2022.105363 (PMC9626688; doi:10.1016/j.isci.2022.105363)

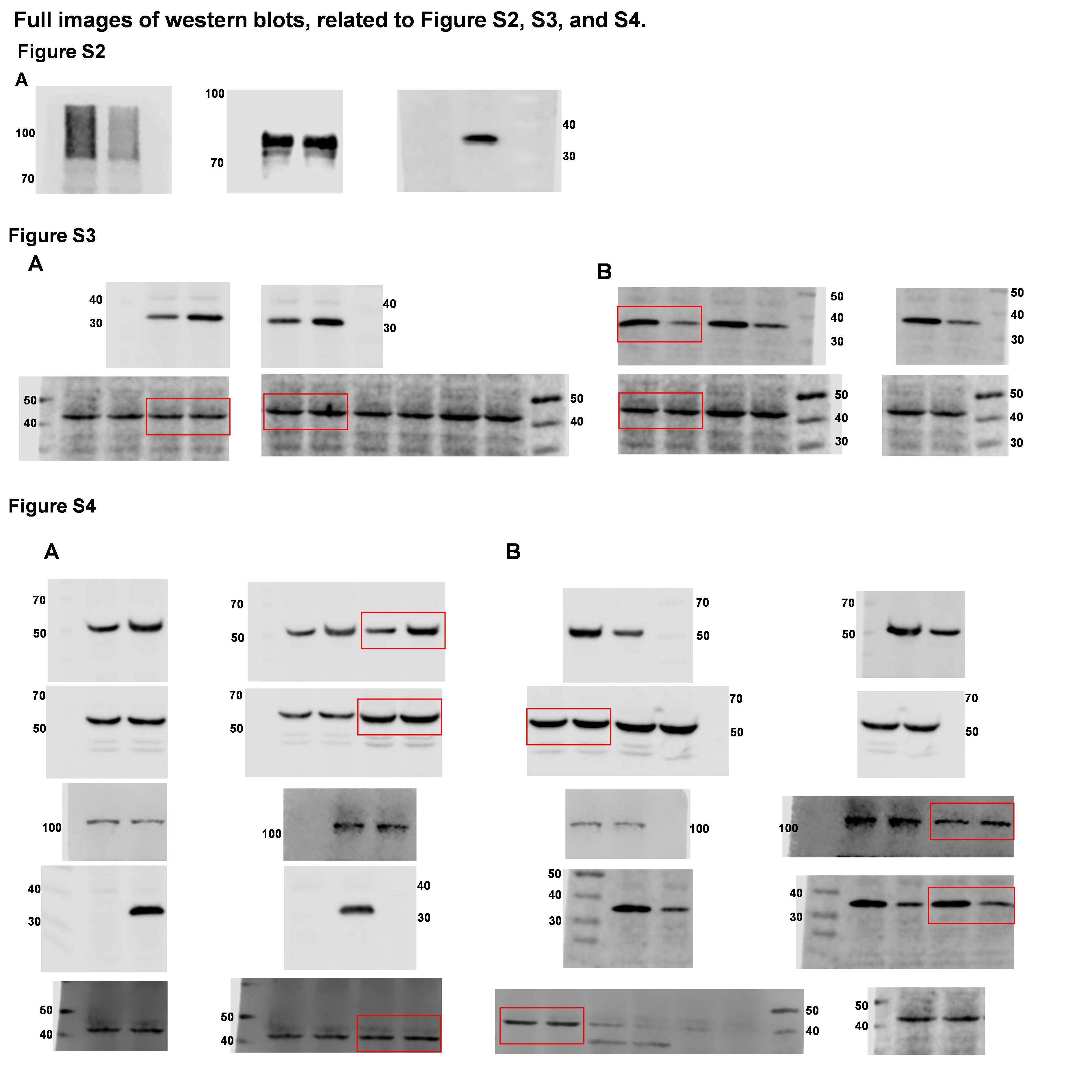

Supplement: Data S2. Full images of western blots, related to Figures 1, 2, 5 and S2–S4 [file mmc3.zip › Full images of western blots, related to Figure S2, S3, and S4..tif]

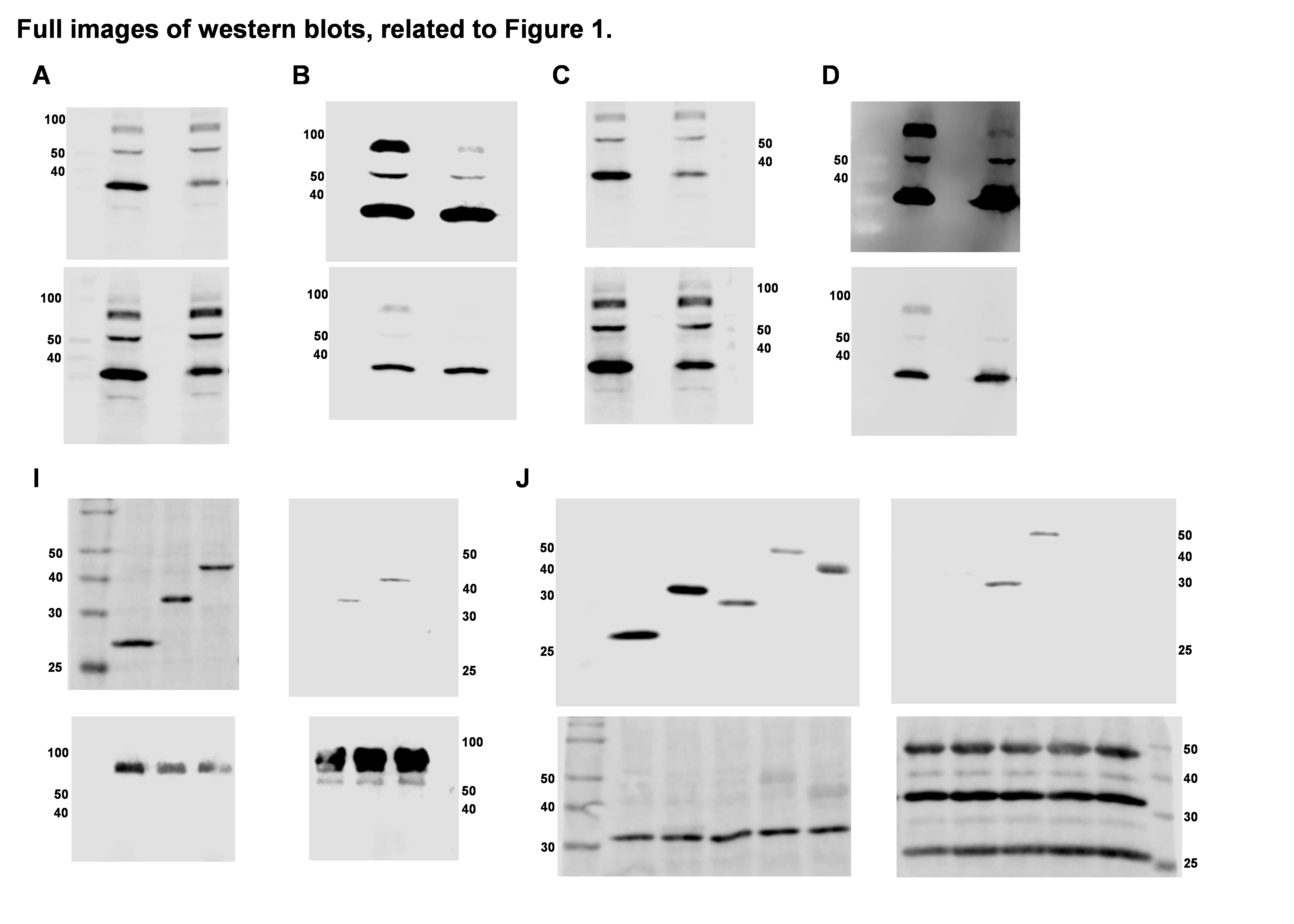

Supplement: Data S2. Full images of western blots, related to Figures 1, 2, 5 and S2–S4 [file mmc3.zip › Full images of western blots, related to Figure 1..tif]

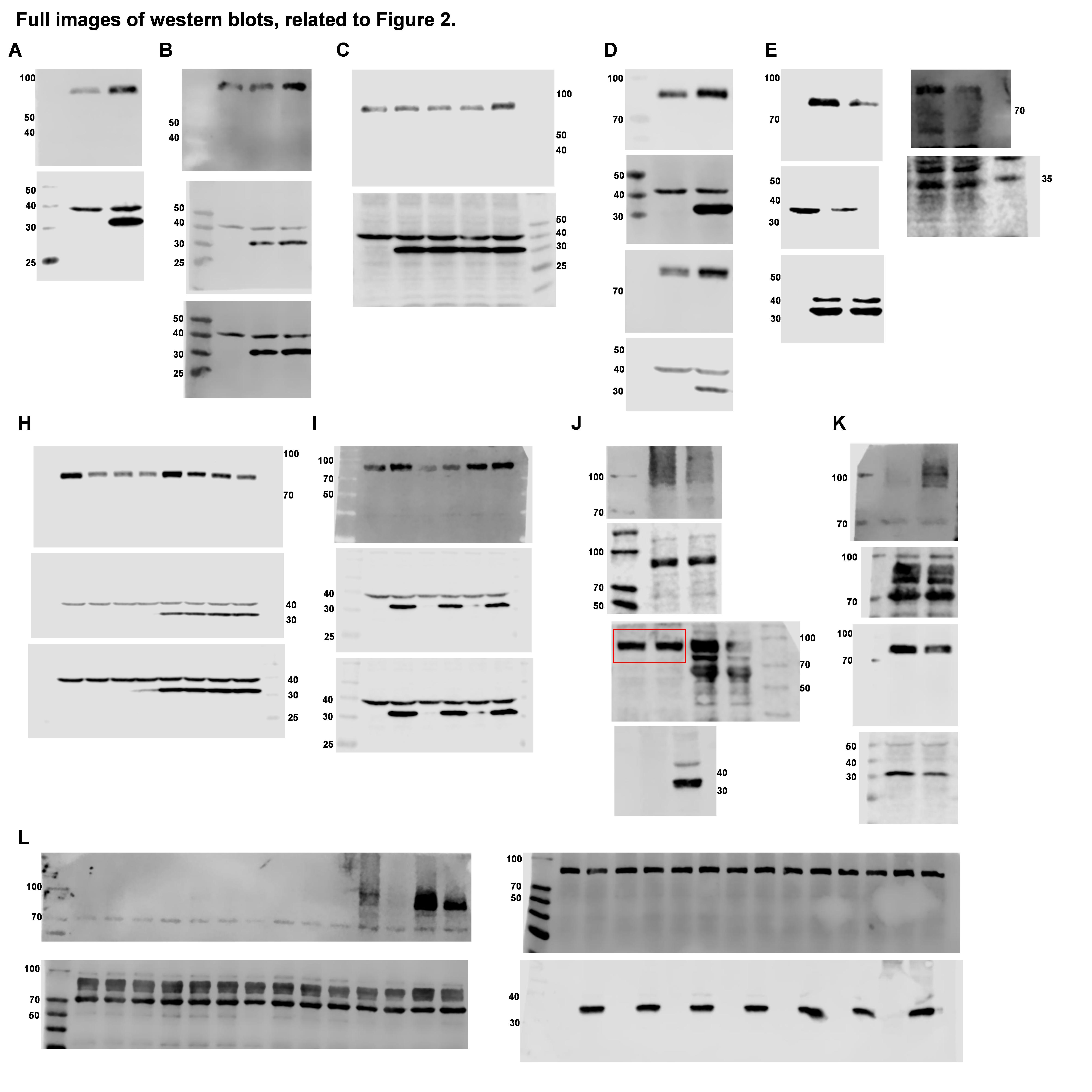

Supplement: Data S2. Full images of western blots, related to Figures 1, 2, 5 and S2–S4 [file mmc3.zip › Full images of western blots, related to Figure 2..tif]

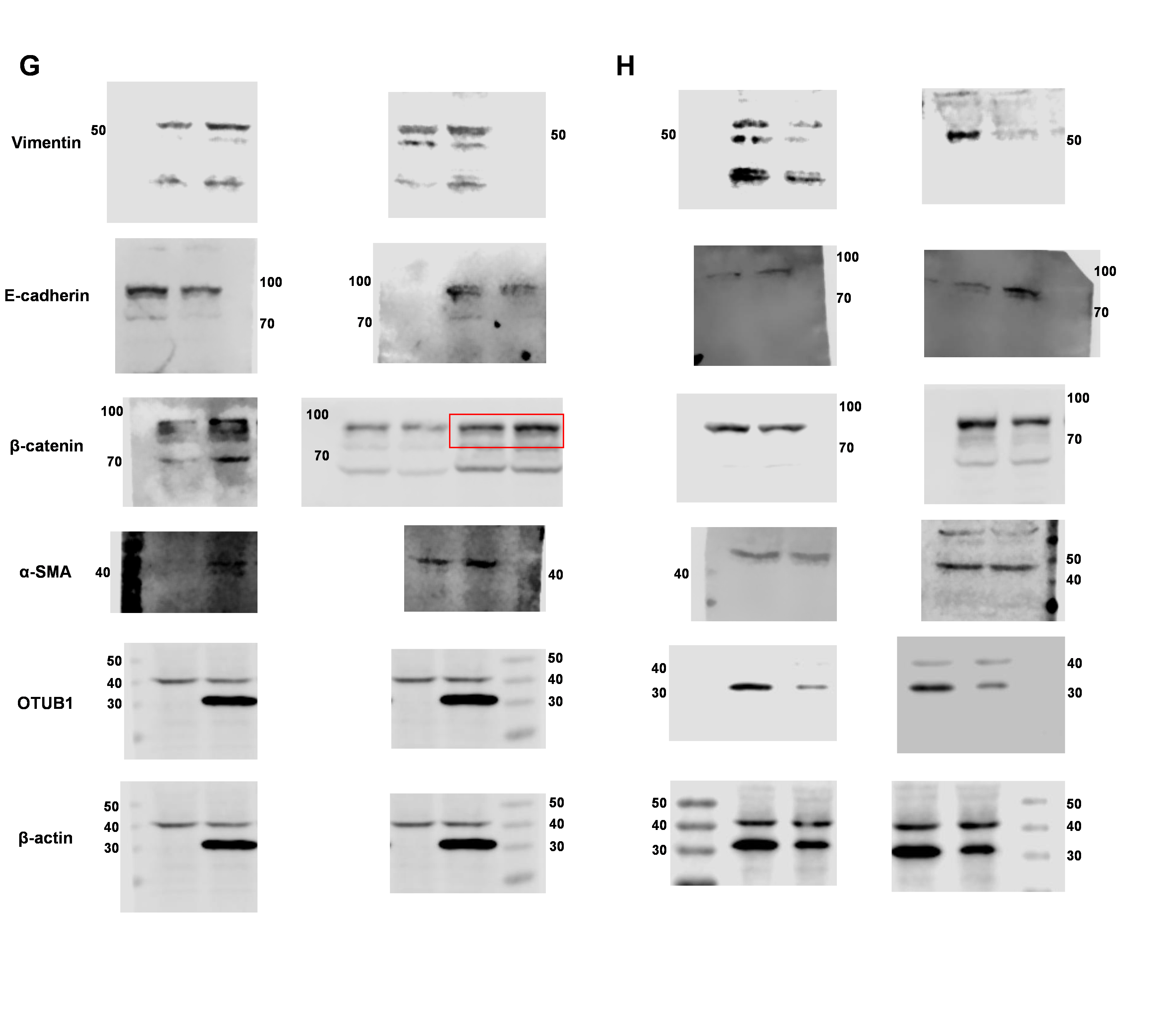

Supplement: Data S2. Full images of western blots, related to Figures 1, 2, 5 and S2–S4 [file mmc3.zip › Full images of western blots, related to Figure 5..tif]
